# Optimal Control of Specification in LPG Blend: A Deep Learning and PSO−Driven Framework for Minimizing Off-Spec Production

**DOI:** 10.1021/acsomega.4c10068

**Published:** 2025-04-08

**Authors:** Aygül Karimova, Güzin Özdağoğlu

**Affiliations:** † Chemical Engineer, Technical Services- Proses Optimization and Monitoring, SOCAR, Siteler, Aliağa, 35800 İzmir, Türkiye; ‡ Dokuz Eylul University, Faculty of Business, Dept. of Business Administration, Division of Quantitative Methods, Central Campus, 35390 Buca, İzmir, Türkiye

## Abstract

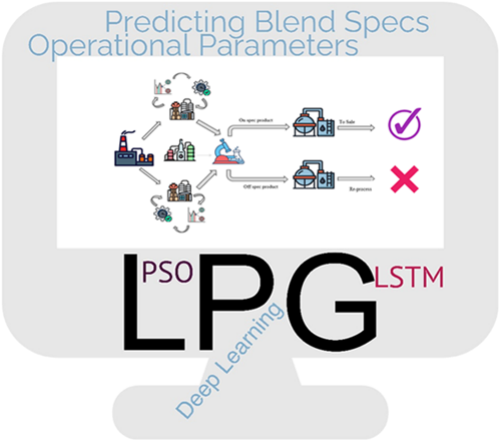

Liquefied Petroleum Gas (LPG) is a crucial energy source, widely utilized in residential, industrial, agricultural, and transportation sectors, where its safe and efficient use relies on accurate product specifications. In the refining industry, LPG is produced in different process units, and final products are blended in LPG storage for sale. Due to changes in the operational parameters of LPG production units, the final product specification can vary. Detection of off-spec production occurs only when the routine sample results are available. However, as production is ongoing, by then, a significant volume of off-spec material may already be blended, posing economic risks such as downgrading or reprocessing off-spec LPG. The annual data set shows that This 10% value corresponds to thousands of tons of product loss and hundreds of thousands of dollars in economic damage. Moreover, failure to meet product specifications can lead to penalties or customer dissatisfaction. To address this challenge, a proactive two-stage approach is proposed. The first stage involves an LSTM-based deep learning model that levers the historical measurement data to predict controllable product specifications within the blending tank. This predictive capability already offers decision-makers significant value by providing early warnings for off-spec formation. However, our research extends this framework by integrating the predictive model with a particle swarm optimization stage. This second stage identifies the optimal operational parameters that can be controlled during the production of LPG in each production unit, ensuring that the off-spec risk in the final tank is effectively mitigated. The methodology uniquely accounts for the differential impacts of identical input variables across various hydrocarbon components, thereby enhancing the precision in capturing the optimal operating conditions for economic savings, ultimately enhancing production efficiency and reducing labor hours. Implementations are limited to the LPG in a particular refinery but can be extended to similar processes. Crude oil types used were not included in this research which can affect the LPG specifications but cannot be manipulated.

## 1 Introduction

LPG is a predominant product, renowned for its versatility as an energy source that supports a wide range of applications due to its exceptional energy efficiency and cost-effectiveness. In residential settings, LPG is indispensable for cooking and water heating, while in transportation, it serves as an economical alternative fuel, particularly favored by commercial vehicles and taxis. Industrially, this product plays a critical role in thermal processing across various sectors, including metalwork, textiles, food production, and ceramics. In agriculture, it facilitates greenhouse heating, powers soil processing machinery, and supports crop drying processes. LPG is also employed in energy generation, particularly in rural regions and during power disruptions, and is utilized for heating and cooling in residential, industrial, and hospitality sectors, including restaurants and food services. Moreover, it is a preferred energy choice for camping and caravan tourism, highlighting its broad utility across diverse sectors.^1^

LPG is produced in refineries as a byproduct during the production of crude oil and natural gas. The production process involves several steps that separate LPG from heavier products like Nafta and lighter ends like refinery off gases.^2^ Operational parameters are employed to regulate the properties of LPG products sourced from various origins, ensuring strict adherence to specified standards. The resulting product is a composite blend of LPGs, meticulously formulated to align with the specific requirements and intended applications of the end users.^3^ This amalgamation process is intrinsically dynamic, influenced by thermodynamic variables such as pressure and temperature, as well as the intermolecular interactions of the hydrocarbon constituents, leading to compositional deviations in the end product from its initial cumulative profile. To guarantee the secure and efficacious utilization of LPG, meticulously defined international standards and requisites delineate its specifications. Critical properties of LPG, such as its compositional integrity, undesired impurities, winter specification, and Sulfur content, must conform to regulatory benchmarks to guarantee both the product’s quality and safety.^4^

While the precise specifications of LPG can vary by nation and intended application, universally recognized criteria typically encompass stipulations regarding the minimum and maximum concentrations of propane and butane, and limitations on sulfur content, odorization levels, and moisture content. However, achieving consistent compliance is challenging due to the variability in the storage and transportation conditions of the hydrocarbon components. Consequently, regular laboratory analyses are employed to monitor these specifications. Given the time-sensitive nature of these analyses, any deviation toward suboptimal quality, or breach of specification (off-spec), can result in substantial product degradation, necessitating reprocessing or leading to significant material losses.

The foremost challenge in this context is accurately predicting the final blend product specifications for storage tanks. Timely and precise predictions can enable preemptive adjustments to avert off-spec scenarios. The secondary challenge involves determining the requisite operational parameters to proactively prevent deviations from the desired specifications. This necessitates an integrated approach, employing two-stage models for effective prediction and optimization, respectively. In the realm of chemical engineering, predictive models have been successfully implemented across various chemical products, and optimization techniques have been explored. However, an integrated approach that consecutively addresses predictive accuracy and operational optimization within a unified model framework remains relatively unexplored. Traditional methods, such as Advanced Process Control (APC) systems, optimize operational parameters but often depend on empirical models that may fail to capture complex relationships between variables.^3^

To address the complex challenges associated with ensuring product specifications in LPG blending, a proactive and innovative two-stage approach is proposed. The first stage employs an LSTM-based deep learning model,^5^ which harnesses historical measurement data to predict controllable product specifications within the blending tank. This predictive capability not only provides early warnings for off-spec formation but also delivers substantial decision-making advantages. Building upon this foundation, different from similar research, our research advances the framework by integrating the predictive model with a metaheuristic particle swarm optimization stage. This second stage determines the optimal operational parameters that can be adjusted during the LPG input phase, effectively mitigating the risk of off-spec conditions in the final tank. In the implementation process, five different LPG specifications are used, i.e., propane content, sulfur content, winter specification, 1.3 butadiene content, and C_5_ content from various sources of production units. Individual models are developed to derive final blend specifications, serving as initial approximations with the optimized parameters of LSTM. PSO is then applied by formulating the LPG specification as an optimization problem.

Notably, this methodology accounts for the varying impacts of identical input variables across different hydrocarbon components, offering a nuanced and precise strategy for capturing optimal production conditions. By facilitating significant economic savings, enhancing production efficiency, and reducing labor hours, this approach represents a transformative solution to a longstanding operational challenge.

## 2 Related Work

### 2.1 Prediction Models

The petroleum industry, known for its vast scale and sensitivity to market fluctuations, relies heavily on accurate predictions for critical tasks such as crude blending, pricing, demand forecasting, and product specification optimization. As one of the primary energy sources worldwide, the sector has increasingly adopted machine learning techniques to enhance operational efficiency, optimize processes, and enable data-driven decision-making. The need for predictive models in this field dates back quite some time,^6,7^ and the models once employed under earlier conditions have now been replaced by more advanced and expanded versions, better suited to the complexities of today’s challenges and conditions.^8,9^ The implementation of deep learning, which particularly supports the infrastructure of artificial intelligence, and the integration of metaheuristic techniques for optimizing these complex operations enable the effective monitoring of production processes and the establishment of precautionary or warning mechanisms against potential risks.^10^

Lababidi et al.^11^ introduced a predictive model to forecast the quality of vacuum gas oil hydrocracking processes. Using experimental data from a pilot plant reactor with the same catalyst as in a local refinery, two sets of runs were conducted: one for the parameter estimation and the other for model validation. The model, based on probability density functions and using SimDist tests, accurately predicts the boiling point as a function of the distilled weight fraction. The model’s simplicity and reliance on specific gravity measurements make it suitable for real-time online analysis, enabling the estimation of conversion rates and product distribution in hydrocracking units. Correa Gonzalez et al.^12^ employed models like Recurrent Neural Networks (RNN), K-Nearest Neighbors (KNN), and Support Vector Regression (SVR) to predict key gasoline properties, particularly the Research Octane Number (RON), demonstrating the ability of machine learning algorithms to capture nonlinear behavior in complex data sets. Similarly, Murty and Rao^13^ developed an Artificial Neural Network (ANN) model to predict RON in gasoline blends, showing superior performance over traditional Multiple Linear Regression (MLR) models. Jiang et al.^14^ introduced a novel dynamic prediction model for pump pressure lift in long-distance petroleum pipelines, combining neural networks, intelligent evolutionary strategies, and an adaptive neural network coefficient matrix. Such studies underscore the capacity of ML to model complex relationships between input and output variables, leading to improved predictive accuracy.

Guleryuz and Ozden^15^ focus on predicting Brent crude oil prices utilizing the LSTM and FBPr models. The research spans 32 years of weekly data and demonstrates the superiority of the LSTM model in accuracy and stability over FBPr, especially in forecasting long-term trends in oil prices. Additionally, Gupta and Pandey^16^ explored crude oil prices by using the LSTM prediction method, comparing different models, epochs, and activation functions to predict crude oil prices. Zhang et *al*.^17^ presented a study on gas concentration models focusing on enhancing safety management in coal mines by predicting gas concentrations. The research utilized an LSTM cyclic neural network, achieving superior performance compared to Bidirection RNN and Gated Recurrent Unit (GRU) models.

Vritska and Simacek^18^ used Partial Least Squares (PLS) and Principal Component Regression (PCR) to predict heavy vegetable oil content in diesel blends, with PLS proving more accurate. Gaspar et al.^19^ examined methods for predicting Reid vapor pressure (RVP) in gasoline-oxygenate blends, highlighting the challenges posed by the nonideal behavior of oxygenates. Additionally, Fatima et al.^20^ demonstrated the effectiveness of Adaptive Neuro-Fuzzy Inference System (ANFIS) over ANN in developing soft sensors for an industrial debutanizer column. Also, Hosseinifar and Shahverdi^21^ introduced a six-point method for predicting distillation curves of petroleum fluids, achieving high accuracy with a third-order polynomial model based on key distillation temperatures and physical properties.

Much of the existing research in the petroleum industry has focused on predictive modeling, such as the use of the LSTM models to forecast production rates, equipment failures, and other time-dependent processes. These predictive models are valuable for anticipating trends and aiding in decision-making. However, while predictions provide insights into what might happen, they do not necessarily guide how to optimize these outcomes. There is a significant gap in the literature concerning the integration of predictive models with optimization frameworks to improve product specifications. This disconnect limits the practical application of machine learning in the industry.

### 2.2 Optimization in Oil and Gas Sector

Optimizing product specifications, particularly in the petroleum industry, involves navigating a complex set of parameters, including chemical composition, processing conditions, environmental factors, and regulatory compliance. The interdependence of these parameters presents challenges in modeling and optimization, especially when faced with multiple, sometimes conflicting objectives. Many studies avoid this complexity, focusing solely on prediction without exploring how to optimize these parameters in practice. This suggests that while predictive models are well-developed, their use in solving real-world optimization problems, such as refining LPG specifications, remains underexplored.

Jonsbraten^22^ applied PSO in Crude Oil Distillation Unit (CDU) optimization, aiming to maximize profitability and minimize CO_2_ emissions. Using a multiobjective PSO algorithm, the study optimized decisions on platform capacity, well drilling, and production strategies. Assareh et al.^23^ applied PSO and GA to forecast oil demand in Iran using socio-economic indicators, with PSO models outperforming GA in accuracy. This research provided valuable insights into strategic planning in the oil sector. The application of Particle Swarm Optimization (PSO) in the petroleum industry, though promising, remains underutilized, especially for prediction and optimization tasks. However, several studies have demonstrated its efficacy in areas like gasoline blending and crude oil distillation. Cheng et al.^24^ used PSO to optimize gasoline blending, addressing the variability in feed properties from different sources. Their nonlinear model, incorporating an RNN, improved the accuracy of the RON prediction for blended products.

Meanwhile, Bayoumy et al.^25^ integrated PSO and GA with Aspen HYSYS to optimize complex systems in gas plants, focusing on the continuous variables often neglected in traditional approaches. Jumaah et al.^26^ focused on CDU optimization, balancing economic gains with environmental impact, and generating Pareto-optimal solutions for product cuts, steam consumption, and CO_2_ emissions. Samad et al.^27^ explored process uncertainties in petroleum refining using artificial intelligence models as surrogates for optimizing process conditions. They employed PSO and genetic algorithms (GA) to improve exergy efficiency, demonstrating that both optimization methods performed comparably under uncertainty. Zhu et al.^28^ explored the relationship between nitrogen diffusion and adsorption in activated carbon through molecular dynamics simulations using a hard sphere/fictitious particle model, focusing on varying porosities and pore sizes and the findings significantly improved adsorption performance by enhancing both the adsorption rate and the saturated adsorption capacity.

The gap between prediction and optimization remains a challenge. Existing studies focus on predictive models without fully integrating optimization. This research addresses the gap by proposing the integration of LSTM networks with PSO for managing LPG product specifications. The LSTM-PSO combination aims to optimize complex variables in LPG production, demonstrating a practical and theoretical advancement in the field. This study not only fills a critical gap in the related literature but also offers direct industry applications, improving product quality and operational efficiency in LPG production.

Combining LSTM and PSO approaches was utilized by Jiao et al.,^29^ which serves as one of the key sources of inspiration for this study. The paper proposed a method for predicting crude oil market volatility based on text mining on an LSTM model and also applied PSO hyperparameters of the LSTM model, further improving its predictive performance. However, the scope and the framework of both models are not similar.

Meanwhile, other metaheuristic algorithms have been developed for different hydrocarbon products. Geng et al.^30^ proposed a multiobjective and customized Gray Wolf Optimization algorithm equipped with an adaptive search mechanism to optimize the parameters within ethylene cracking furnaces—one of the most critical devices in ethylene and propylene production. Their algorithm was designed to enhance efficiency by analyzing the interactions among the components processed within the furnace. Through its adaptive search structure and additional regulatory functions, the proposed approach mitigates the limitations of traditional metaheuristic algorithms, ultimately achieving a significant increase in overall efficiency. Graph-embedded deep learning network structures are increasingly employed to detect anomalies in time series data. Recently, novel approaches integrated into conventional frameworks have demonstrated significant improvements in anomaly detection accuracy. Zhang et al.^31^ successfully applied a graph-embedded deep learning process, combined with an optimization framework, to the fluid catalytic cracking process^32^. Their work stands out as one of the rare studies that simultaneously utilize prediction and optimization models to enhance chemical processes effectively. Their study has a predictive model to detect anomalies, and the integration of the optimization process improves the strength of the detection model. Our study differs from Zhang et al. in that we use optimization again for the process using the predictions to find values of the operation parameters, therefore, the optimization phase serves as a decision support for the process supervisors and the executive team.

## 3 Methods

### 3.1 The Problem Definition and Modeling Framework

This study specifically focuses on LPG due to its complex nature within refinery operations. Unlike some other refinery products with more standardized specifications, the final LPG product of refinery is often blend of LPG products or byproducts of different process units such as Catalytic Cracking units, Saturated and Unsaturated gas plants. As a result, the specifications of LPG can vary significantly based on the composition of these byproducts and the specific operational parameters involved in its production. The variability in LPG specifications is further compounded by fluctuations in the operational parameters such as temperature, pressure, flow rates, and catalyst activity. Additionally, the blending of different streams of crude oil and intermediate products can introduce additional variations in LPG composition and properties. These dynamic factors make it challenging to consistently produce LPG that meets all specified criteria, leading to occasional instances of noncompliance with standards and subsequent economic losses.

**Figure 1:**
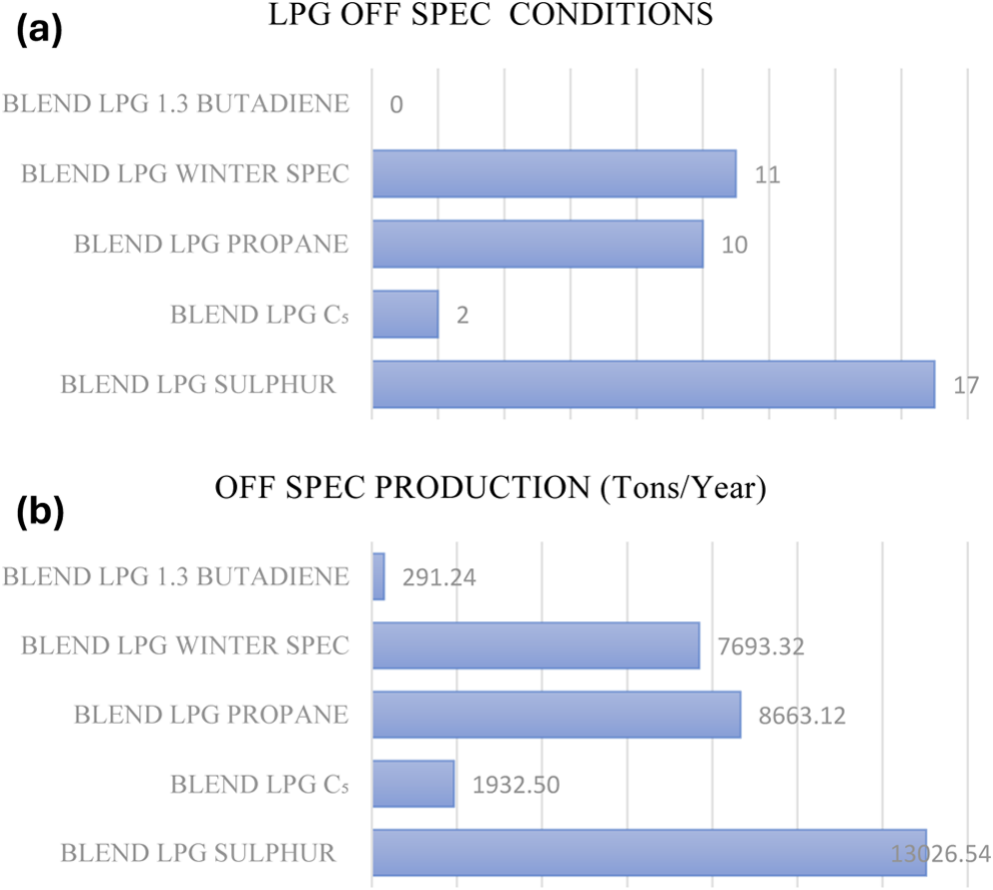
(a, b) Off-spec production distributions.

The volatility in the composition of LPG production in refineries poses a challenge to ensuring that the product meets the required specifications and thus consistent product quality. LPG and its components, originating from different feed lines, converge in the blending tank to form the final product. Due to the influence of environmental factors on the feed lines, the operational characteristics of LPG components, and the interactions among these variables, the properties of the product within the tank cannot be precisely determined in advance. Consequently, the results of time-consuming tests must be awaited. For instance, the procedure of sampling, sending samples to laboratories, conducting analyses, and reporting results typically takes more than 4 h, highlighting the urgency and importance of efficient prediction and optimization strategies. When test outcomes reveal out-of-specification values, immediate corrective actions cannot be implemented, resulting in the continued production of undesired product blends during the testing intervals. Within the collected data set, approximately 10% of the cases were identified as noncompliant with specifications. The annual frequency distribution of these instances is presented in Figure 1a, while the corresponding total production value is illustrated in Figure 1b. Although the number of noncompliant cases appears relatively low, the associated production of thousands of tons of defective products has a significantly negative impact on the efficiency of the process. Off-spec LPG must be evaluated either as fuel gas or reprocessed, both of which incur additional costs. In this regard, the economic value of these noncompliant cases averages around $800,000, highlighting their substantial financial implications.

Therefore, traditional methods often rely on reactive measures, based on postproduction testing, which can lead to inefficiencies, waste, and higher operational costs. To address this challenge,^30^ a proactive two-stage approach has been developed within the framework of the flowing questions:

- How can an effective model be developed and utilized to accurately predict LPG specifications in the blend?
- Can an optimization model be developed for operational parameters, based on predicted product specifications, to achieve optimal conditions for continuous on-spec product production?

Specifically, the proposed model aims to optimize the production process by starting with the prediction of the values of LPG components. In the first stage of the model, a deep learning-based prediction method is presented that calculates the final values before shipment. Although this prediction model enables the early detection of results that do not meet product specifications, without waiting for laboratory analysis results, there are challenges in taking proactive actions promptly.

To overcome these issues, the results produced by the prediction model are utilized as an input set to an optimization model in the second stage. This model aims to prevent the formation of off-spec products by determining the optimum operational parameters. The complexity of the optimization model increases due to the diversity of sensitivities of different components to operational parameters and the conflicting effects of these parameters on each other. This research demonstrates the applicability and effectiveness of the proposed model using real production data and contributes significantly to increasing efficiency and reducing production costs in industrial processes.

The proposed two-stage integrated model represents a proactive approach, combining predictive analytics with advanced optimization techniques to pre-emptively adjust operational parameters, ensuring product quality, and operational efficiency. The first stage utilizes deep learning techniques, specifically LSTM networks, to accurately predict the final composition of LPG before shipment, based on data from various sources. The LSTM model’s ability to capture temporal dependencies in time-series makes it particularly suited for forecasting the complex behavior of LPG compositions over time. In the second stage, the research leveraged a combinatorial algorithm, namely Particle Swarm Optimization (PSO), to fine-tune the operational parameters in response to the predicted LPG component values. PSO’s capacity to navigate multidimensional search spaces effectively allows for identifying optimal settings that minimize the risk of producing off-spec products, thus addressing the limitations of the predictive model by enabling timely and informed adjustments to the production process.

**Figure 2:**
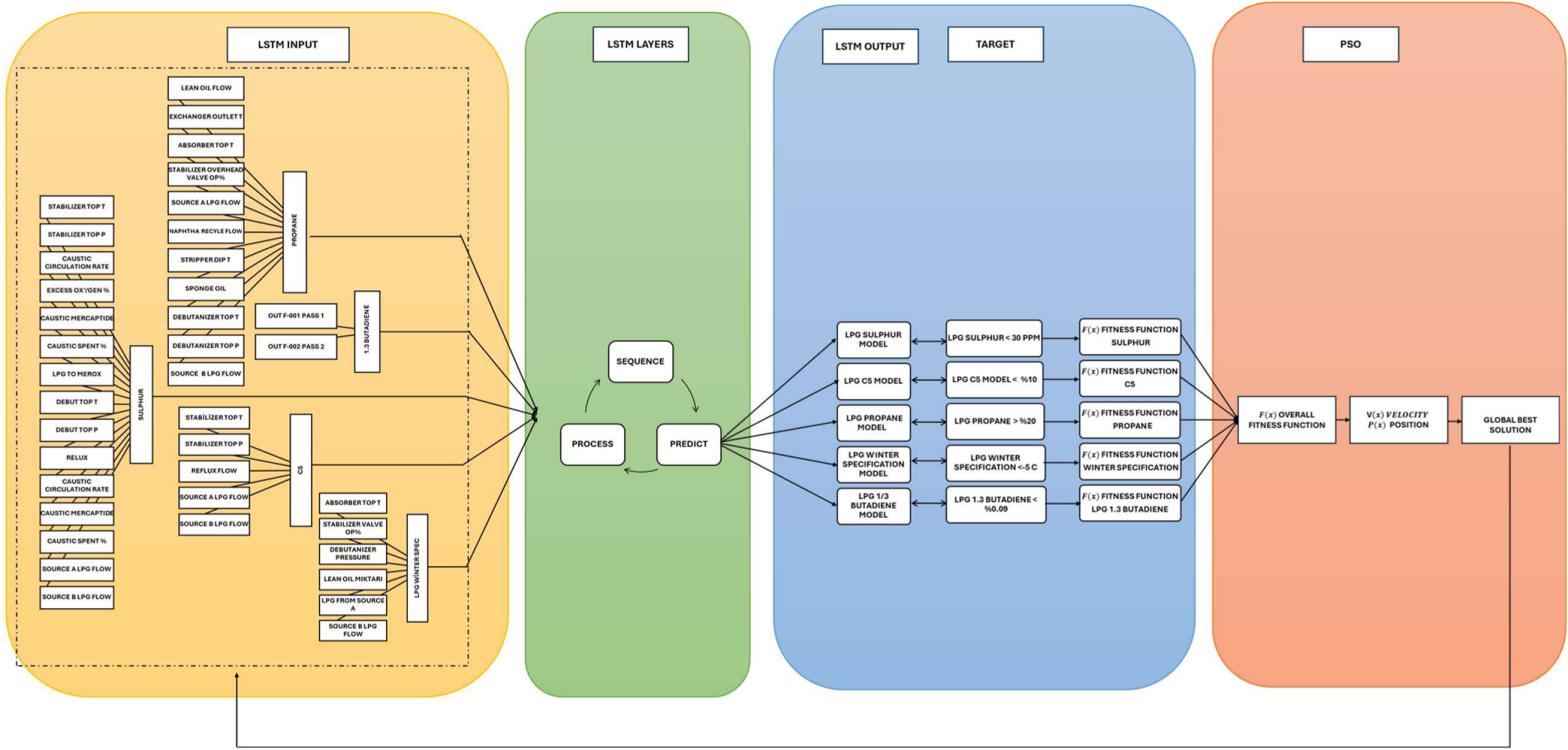
LSTM + PSO framework.

The diagram in Figure 2 illustrates the methodology of integrating the PSO algorithm with an LSTM network for optimizing and forecasting refining operations. The LSTM model is designed with three primary layers: processing, sequence generation, and forecasting, each responsible for handling time-series data effectively. Input parameters in this model include variables such as column temperature, column pressure, composition, reflux, and flow. While the diagram shows a few examples of these parameters, the developed model encompasses a much larger data set—117 input parameters with 1090 data points. These input variables are critical to both the PSO and LSTM models, as they represent key operational parameters that influence LPG product specifications.

The iterative process continues until the PSO algorithm converges on a global best solution, which represents the optimal set of input parameters for the LSTM model to produce predictions that meet the target LPG specifications. The system ensures that the predictions closely align with the target values, refining the operational parameters to achieve the desired performance in real-time. This approach not only improves the flexibility and sustainability of LPG processing but also lays the groundwork for applying similar methodologies to other complex industrial systems. By addressing the key operational challenges, this research sets a precedent for optimizing various processes that involve time-dependent data and multiple interrelated parameters. The in-depth methodology developed in this research seeks to reveal the intricate dynamics that govern LPG production, creating a robust foundation for future advancements in predictive models and optimization strategies. The resulting framework not only enhances production efficiency but also offers a replicable model that can be adapted across various sectors of the chemical and refining industries.

### 3.2 Data Collection

Before the data collection, explaining the LPG production^2^ process is crucial for understanding the dependencies and interactions among various specifications and operational parameters within LPG production units. Prior to the initiation of data collection, a detailed analysis is conducted to comprehend how each specification of LPG is interdependent. From a process engineering perspective, the operational parameters that could potentially influence these specifications are identified. This includes an examination of both the positive and negative impacts these parameters might have on LPG specifications. Additionally, the analysis determines the interdependence among the specifications themselves, exploring whether changes in one specification could inadvertently affect others.

**Figure 3:**
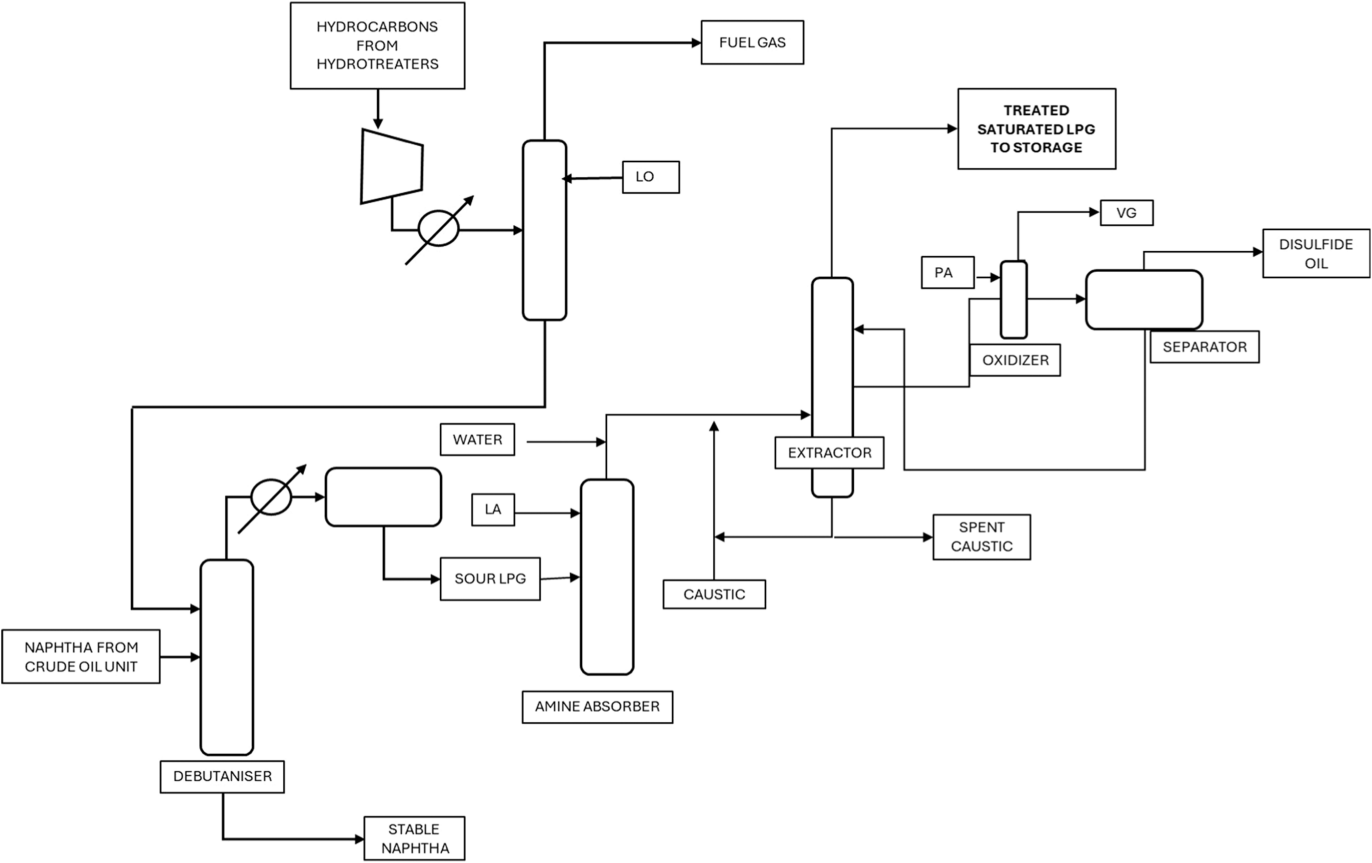
LPG from source A—saturated Merox process flow diagram.

**Figure 4:**
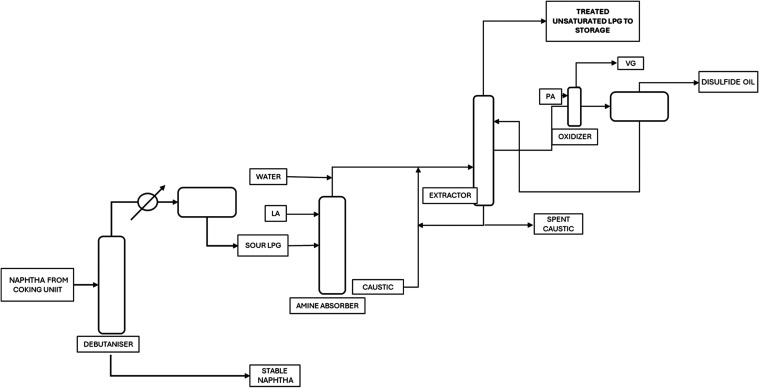
LPG from source B—Unsaturated Merox process flow diagram

Data is systematically gathered over a one-year period, emphasizing both upstream and downstream unit parameters to ensure a comprehensive understanding of the entire LPG production process. To account for the inherent time lag between the sampling analysis and the recording of operational parameters, data collection is synchronized across all specifications and units using identical time steps. This approach facilitates a more accurate and meaningful analysis of the data, allowing for the identification of temporal patterns and correlations. The process flow diagram of typical saturated and unsaturated LPG production processes (LPG coming from source A and LPG coming from source B) are shown in Figures 3 and 4. LPG from source C was not a part of this research, so data from source C is directly used in overall LPG blend calculations.

The collection of data related to LPG production from sources A- Saturated Merox unit, B- Unsaturated Merox unit, and C- CCR unit is meticulously organized according to the specific LPG specifications. Operational parameters with the potential to influence the specifications are identified based on process engineering insights such as temperature, pressure, and flow rates at various points in the production process. Data collection efforts are synchronized across all parameters and specs, employing identical time steps to ensure consistency. This attempt is particularly important for capturing the effects of operational changes on LPG specifications on time. Accordingly, an analysis is conducted to explore the interdependence among different LPG specifications therefore this analysis involves assessing how variations in one specification might impact others, contributing to a holistic understanding of the production process. The selected parameters are critical for understanding the complexities of LPG production, allowing for a comprehensive analysis of how each factor contributes to the efficiency, quality, and reliability of the process.

The difference between the Saturated and Unsaturated Merox process is mainly the composition of LPG. In Unsaturated Merox unit the feed is cracked hence unsaturated and % of C_4_ is less than C_3_ hydrocarbons, while in the case of Saturated Merox, LPG composition is saturated and % of C_4_ is higher than C_3_. Although the process flow diagrams of each source of LPG are similar due to the nature of feed composition, the specifications vary, and the control of these operating parameters also changes accordingly.

Variations of LPG specifications are described below:

- LPG Sulfur: LPG undergoes treatment in Merox units, where caustic is utilized to treat Sulfur in the form of mercaptans. The parameters vital to the LPG Sulfur treatment process include caustic flow, air flow, and Merox catalyst. Additionally, the temperature of the caustic, LPG, and caustic specifications are important factors. Therefore, data was collected for these variables in each LPG source. Separation from Heavier Petroleum Products: Before reaching the Merox unit, LPG is separated from heavier petroleum products, such as naphtha. LPG Sulfur content tends to increase in heavier hydrocarbons. Although the composition of LPG primarily includes butane and propane, impurities such as LPG C_5_ are also present. Therefore, data was collected for the content of C_5_ hydrocarbons (pentane, pentene, isopentane, and others), as increasing their presence also increases LPG Sulfur content. The data set also includes separation column operating parameters such as pressure, temperature, and reflux ratio.
- LPG Winter Specification: The LPG winter specification is regulated by the vapor pressure of light ends in the product, mainly propane and ethane. During winter, lighter hydrocarbons need to be maximized in the LPG composition to maintain a minimum vapor pressure of −5 °C at 150 kPa, as required by European standards (Grade B LPG specification). The separation column’s temperature and pressure are managed to achieve this. Since lighter hydrocarbons have lower condensation temperatures compared to heavier ones, the temperature in the column is minimized to condense them into LPG. Alternatively, pressure can be increased. Hence, the data set includes column operating parameters.
- LPG C_5_: The regulation of C_5_ hydrocarbons in the column is based on standards, with the percentage typically ranging from 5% to 8%, depending on LPG/naphtha price differences. As increasing C_5_ content can result in higher Sulfur content, both specifications are controlled based on their importance. The data set for each LPG production source includes columns for operating pressure, temperature, and reflux ratio. The LPG sources are designated as A, B, and C. Sources A and B are the subjects of prediction and optimization, while Source C is excluded from the research and only used in blend calculations. The sources are defined as follows:
  - Source A: LPG from the Crude Atmospheric Distillation Unit
  - Source B: LPG from the Coker Unit
  - Source C: LPG from the Catalytic Reforming Unit
- LPG Propane: Although LPG propane composition primarily depends on the source’s production, as well as crude oil API and composition, it is possible to maintain LPG propane content above the minimum standard (>20%). LPG from unsaturated Merox units, where LPG comes from FCC and coking units, consists of higher propane and lower butane content. On the other hand, saturated LPG from crude has lower propane content. To meet the propane content standards for LPG blend, the condensation process is increased. Unlike the winter specification, this is not a seasonal requirement, so it needs to be maintained consistently during summer. The data set includes relevant parameters to ensure compliance.
- LPG 1.3 Butadiene: This spec is highly important because when using LPG as a fuel the component 1.3 butadiene, which is mainly included in unsaturated LPG, may damage both human health and the environment. As the origin of 1.3 butadiene cocker unit, which is the product of cocking, parameter of managing this spec is heater temperature. However, it is also important to keep heater temperatures in a way that does not lose the main product yields in the unit as well. The product specifications in question can be influenced either directly or inversely by changes in operational parameters. Moreover, a single parameter may impact multiple specifications in different directions, creating trade-offs for the operational parameter values and highlighting the necessity of optimization. Since the specification values in blending tanks, where components from LPG sources are combined, cannot be determined additively and interactions further complicate the outcomes, there arises a need to predict the in-tank values accurately.

Under these conditions, the proposed approach employs an initial prediction phase to estimate the specification levels in the blending tank based on operational parameters. Subsequently, an optimization process is implemented, considering deviations of these values from the upper or lower specification limits, to identify parameter settings that prevent off-spec production. This approach is methodical and data-driven, enabling seamless application across different data sets without significant challenges. In the prediction process, all measured inputs influencing the specifications were utilized. However, during the optimization phase, only those inputs that could be adjusted or controlled were incorporated into the optimization process. The data was provided under specific constraints and in accordance with an agreement. While the raw data is not open for sharing, permission has been granted to publish the analyses and findings for academic purposes.

### 3.3 Prediction Model with Deep Learning (LSTM Model)

Deep learning, a subset of machine learning, has emerged as a powerful approach for tackling complex problems across various domains by mimicking the human brain’s ability to learn from data. At its core, deep learning relies on artificial neural networks with multiple layers of interconnected nodes, allowing for the automatic extraction of intricate patterns and features from vast amounts of data.^5,45,47^

LSTM in deep learning, a type of RNN, was designed to overcome the vanishing gradient problem and learn long-range dependencies.^33^ LSTMs incorporate a cell state for storing long-term information, along with input, forget, and output gates to manage the flow of information.^34^ The hidden state serves as the output at each time step, contributing to pattern recognition and feature extraction.^35^

Activation functions play a crucial role in neural networks by introducing nonlinearity, enabling the model to learn complex relationships between inputs and outputs.^34^ Key activation functions include linear, exponential, exponential linear, tanh, sigmoid, ReLU functions.^36^ In this research the most used activation function for LSTM prediction model development were mainly *linear*, *exponential*, and *tanh* functions since among the others these functions performed better (eqs 1−3).

- Linear Function:
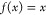
1
- Exponential Linear:
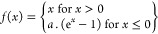
2
- Tanh Function:

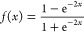
3

RNNs are highly effective for handling sequential data coming from process outcomes in chemistry. Even deep learning such as RNN models inherently possesses complex structures, managing this complexity and making the model practical become more feasible with the help of coding libraries, e.g., TensorFlow, Keras, and PyTorch.^37^ In this study, Python libraries are utilized to transform the model into a practical and implementable procedure. The choice of framework depends on the project, data characteristics, and user preferences.^33,38,39^

To implement an LSTM model, data preparation is the key point. Following the initial data preprocessing, the data is standardized to a common scale in the range of [0,1] using interval normalization,^40^ then decomposed into training (80%) and testing (20%) sets. A function is then created to generate sequences of data points to help the model learn patterns. Using Keras’ Sequential model, the LSTM architecture is built with one or more LSTM layers and a Dense layer for the output. The Adam optimizer and mean squared error loss are used, ideal for regression problems.^41^ After training the model over multiple epochs, predictions were made, and results were inverse-transformed to the original scale. The example provided in this study serves as a foundational implementation, with room for customization based on the data set and problem specifics. Hyperparameters such as the number of LSTM layers and units are adjusted to optimize performance.^40^ This methodology outlines the practical application of LSTM networks for the time series prediction,^42^ forming an essential part of the research’s analytical approach.

**Figure 5:**
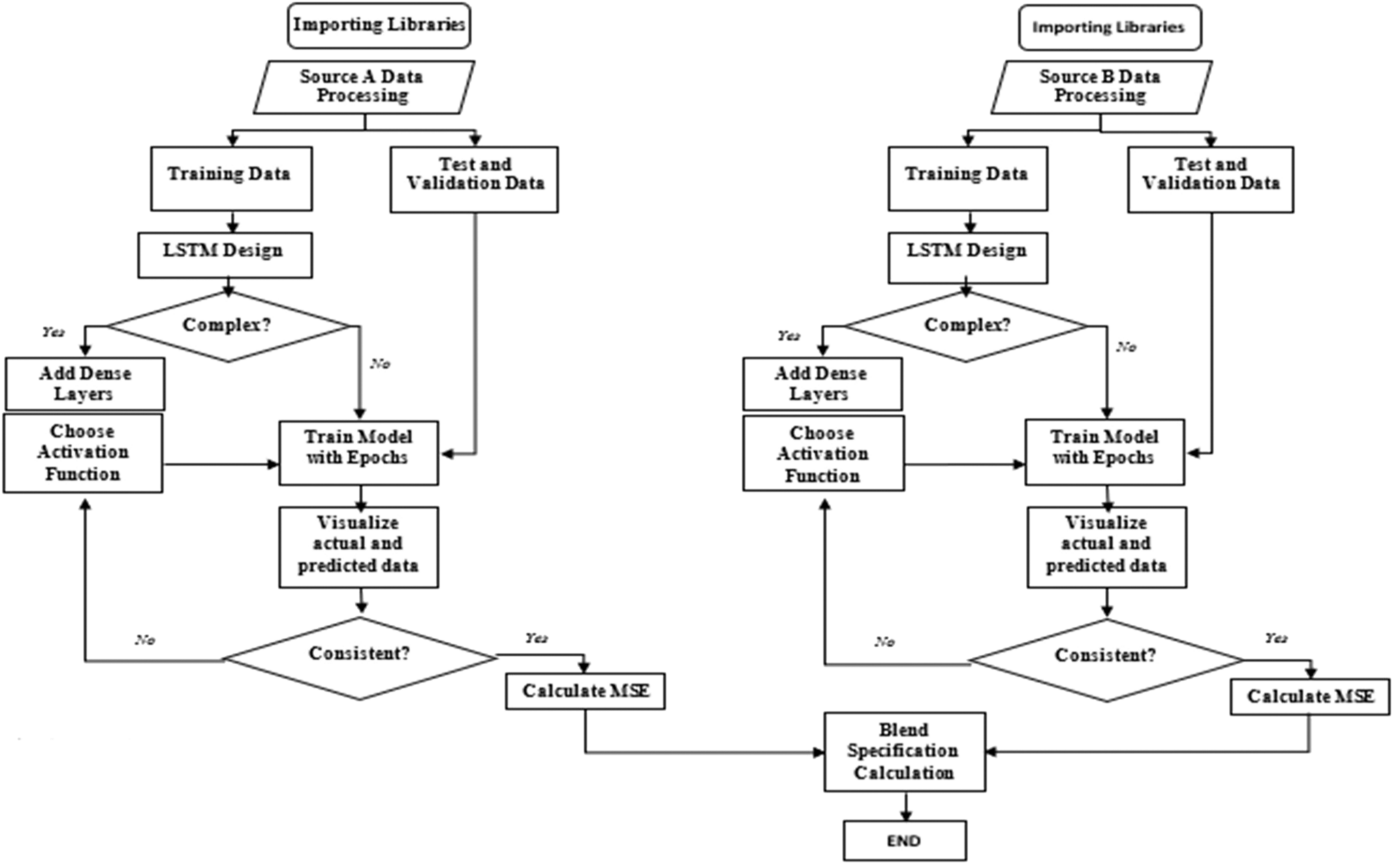
LSTM model-building steps.

The model is trained on the preprocessed data, and the training process is monitored by evaluating loss and performance metrics. The model is then evaluated on the test set, using mean squared error (MSE) to assess performance. Fine-tuning and parameter optimization are carried out to improve the prediction model by adjusting hyperparameters and architecture. Figure 5 illustrates key decision points in the model-building process, suggesting a revision of the activation function if significant discrepancies between actual and predicted values are observed.

### 3.4 Optimization of Operational Parameters (PSO Model)

PSO is a metaheuristic algorithm inspired by social behavior, particularly the movement of bird flocks or fish schools, introduced by Eberhart and Kennedy in 1995. It simulates the collective intelligence of a group of individuals (particles) moving through a solution space, where each particle represents a potential solution to an optimization problem.^43,44^

PSO includes the following key components:

- Particle Representation: Particles are characterized by position and velocity vectors in the search space, representing possible solutions. In this study, each potential solution vector is a representation of the PSO model.
- Position and Velocity Updates: Particle positions are updated based on their current velocity, personal best, and global best-known positions^45^ as indicated in eq 4.
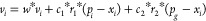
4where,
  *v*_*i*_: Velocity vector of particle *i*
  *x*_*i*_*:* Position vector of particle *i*
  *p*_*i*_*:* Personal best position of particle *i*
  *p*_*g*_*:* Global best position found by any particle in the swarm.
  *w:* Inertia weight, a constant balancing the trade-off between exploration and exploitation.
  *c*_1_, *c*_2_*:* Cognitive and social coefficients, controlling the influence of personal best and global best, respectively.
  *r*_1_, *r*_2_*:* Random values between 0 and 1.
- The fitness function evaluates the quality of a particle’s solution and guides the swarm toward the optimal solution by quantifying how well a solution meets the problem’s objectives. It returns to a numerical value, helping particles adjust their positions accordingly.^43,46^ This research adopts a fitness function that covers all specifications and operational parameters together with the intervals. The fitness function was formulated to minimize the absolute differences between their values and their corresponding reference ranges. Therefore, each component of the fitness function is dependent on the spec characteristics. eq 5 exemplifies only one of the components belonging to the Sulfur spec. This is a small part of the fitness function for Sulfur specification of LPG. Here only one parameter − Stabilizer column operating temperature is given however in the rest of the code all 14 parameters and their reference and scale absolute differences are included.
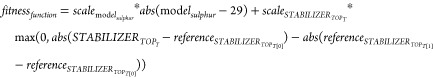
5
  In the formula, only one parameter- Stabilizer top temperature is shown, other parameters are also included in the fitness function used in the algorithm.
- Key Factors are Inertia Term which balances exploration and exploitation, and Cognitive and Social Terms which direct particles toward their personal best and the swarm’s global best solutions.

**Figure 6:**
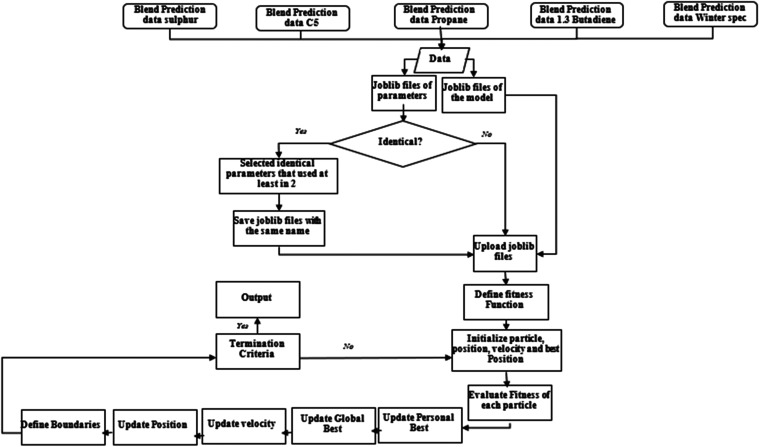
PSO model-building steps.

Prediction serves as the foundation for optimization, as understanding how parameters affect specifications is essential. This phase starts by defining parameters for optimization, creating *joblib* files for the prediction models to be able to call and execute them in optimization model to avoid reloading models, and selecting relevant parameters for optimization. Next, specifications for optimization are identified, using blend prediction models and saving training data. An objective function is then defined, typically minimizing error based on model predictions. The search space is established by identifying tunable parameters and their ranges, with a scaling factor in the fitness function balancing the optimization. A swarm of particles is initialized, each representing a potential solution, with positions and velocities set randomly or strategically. The fitness of each particle is evaluated based on the objective function. Velocities and positions are updated using formulas that incorporate personal and global best positions. The process is iterated until a termination condition is met, tracking the best solution (global best) throughout. The optimized solution is then evaluated for performance improvements and applied to the machine learning model for rerunning predictions. This framework guides the use of PSO following predictions, with variations depending on the specific problem and PSO algorithm (Figure 6).

In the integrated approach, PSO plays a vital role in optimizing the input parameters. It adjusts and updates these parameters by refining the velocity and position of potential solutions in search of the best global result. The fitness function represented by *F*(*x*) evaluates the predicted outputs from the LSTM model against specified target values, such as

- LPG sulfur content: Target <30 ppm
- LPG C5 (Pentane): Target <10%
- LPG propane: Target >20%
- LPG winter specification: Target < −5 °C
- LPG 1,3-Butadiene: Target <0.09%

It is possible to explore different variants of PSO. In particular, the velocity component can be tailored according to the specific type of problem being addressed.^48^

## 4 Results and Discussion

### 4.1 LPG 1.3 Butadiene Prediction

In the prediction model for 1.3 butadiene, data from Unit A, where 1.3 butadiene is present in unsaturated LPG production, was uploaded to the modeling framework and eight selected parameters along with actual 1.3 butadiene values were analyzed. Since 1.3 butadiene is measured through laboratory analysis, a time lag between analysis and data was considered, as the effect of parameters on butadiene becomes visible after 8−12 h. A total of 1081 data points per parameter were included in the analysis. The data set was divided into training, testing, and validation sets to evaluate the model’s performance for both short and long-term predictions. A window size of 8 h was chosen, which was sufficient given the time span of the data. In the LSTM prediction model, this window size refers to the number of previous time steps used to predict the next time step. For example, heater temperature values from the previous 8 h were used as input to predict the temperature for the next day, with the window sliding by 1 day at a time.

The TensorFlow framework, integrated with Keras, was used for developing the procedure of the solution framework. The MSE metric was employed to evaluate performance, and the Adam optimizer, which adjusts the learning rate dynamically, was used for training. The Model Checkpoint callback was implemented to save the model weights during training based on the best validation loss. After importing the necessary libraries, a Sequential model was chosen with an 8-h window size. The LSTM layer had 64 units, and after experimenting with activation functions, it was found that the *Tanh* function worked best for predicting 1.3 butadiene.

**Figure 7:**
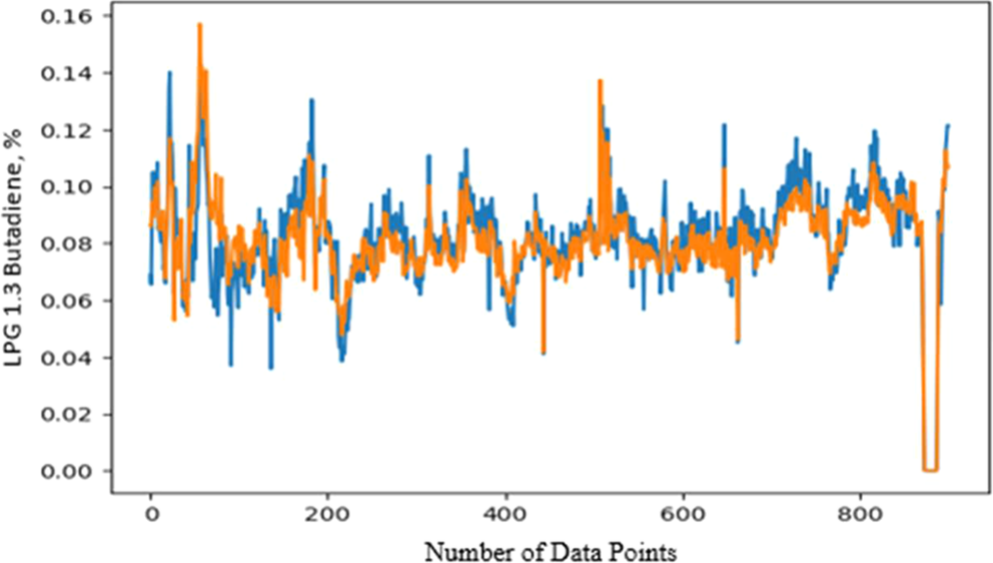
LPG blend 1.3 butadiene specification.

The model also included two fully connected dense layers: one with eight units using the Tanh activation function, and the final layer with a single unit and a linear activation function for the output. The model trained over 10 epochs, meaning the entire data set was processed 10 times during training. The best model, with the lowest MSE, was saved. Finally, the predicted values were plotted to compare them with the actual values. The result shows a value of 0.00156, which is small and acceptable. So, minimizing MSE function makes our model more accurate. As mentioned above, 1.3 butadiene is an impurity coming from downstream unit, mainly cocker unit, so it is by product of cocking process. Mainly it is in unsaturated LPG, so, only source A includes this impurity, while other sources, 1.3 butadiene is 0. So, blend specification is calculated both actual and predicated values of 1.3 butadiene (Figure 7). The blue line refers to the actual data and orange line refers to the predictions generated by the model.

For the other specifications, blend indicators are calculated based on eq 6.
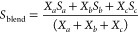
6where

*S*_blend_: Blend product specification

*S_a_*: LPG specification from source A

*S_b_*: LPG specification from source B

*S_c_*: LPG specification from source *C*

*X_a_*: LPG flow rate from source *A*

*X_b_*: LPG flow rate from source B

*X_c_*: LPG flow rate from source *C*

If any of the blend specifications are out of specifications, the product should be sent to an off-spec storage, and operational parameters should be analyzed and optimized to get the product back on spec.

### 4.2 LPG Winter Spec Prediction

Although the LPG winter specification is typically relevant during colder months, the data set covers the entire year to allow the model to analyze the long-term effects of operational parameters such as temperature and pressure. The prediction model for Unit A’s LPG winter specification was developed using 23 parameters, along with the actual values of the winter spec (minimum vaporization temperature at 150 kPa). The data set spans one year, with data points taken at 8 h intervals.

To assess both short-term and long-term prediction performance, the data was split into training, testing, and validation sets, with the training set focused on long-term data. The Keras TensorFlow framework was utilized for model development, and essential libraries for the sequential model, Adam optimizer, as well as MSE for loss evaluation, were activated. The model was designed with an LSTM layer containing 64 memory units, followed by two dense layers. The first dense layer has eight neurons with an exponential activation function, while the second layer has a single neuron with a linear activation function. Various activation functions were tested, and the combination of exponential and linear functions was found to provide the best prediction results. Next, a prediction model for the LPG winter specification of LPG from Source B was designed using operational parameters from Unit B. As previously mentioned, LPG from Source B is rich in propane, unlike Source A, which is richer in butane. The winter spec (minimum vaporization temperature at 150 kPa) depends on the content of light hydrocarbons. The higher the concentration of lighter hydrocarbons, the lower the LPG minimum winter specification, which positively impacts the winter specification. For the prediction model of the winter spec for LPG from Source B, the data set included nine parameters and the actual values of the winter spec. One year of data was used, with an 8-h interval between each data point. The reduced number of parameters compared to the model for LPG from Source A is since Source B consists of unsaturated LPG from a single source, while saturated LPG from Source A involves multiple steps before reaching its final form, which requires more parameters in the model.

**Figure 8:**
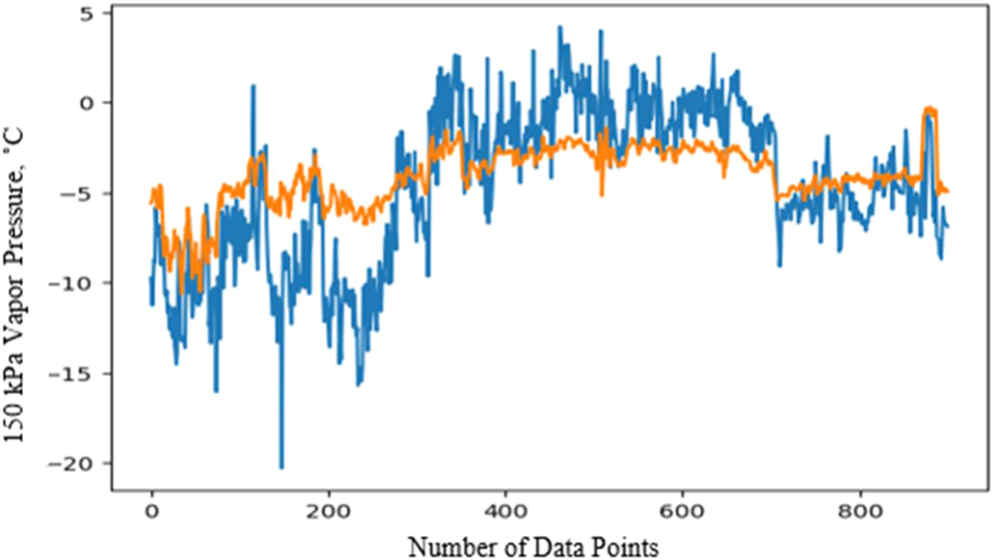
LPG blend winter specification.

The same model design used for the winter spec prediction of LPG from Source A was applied here. This included the LSTM layer with 64 memory units, two dense layers—one with eight neurons using the exponential activation function and one with a single neuron using the linear activation function. After testing different configurations, the exponential and linear activation functions again proved to be the best combination for accurate prediction. The MSE was calculated at 0.38, which was the lowest compared to other activation functions tested, confirming the model’s efficiency for LPG from Source B. The LPG winter specification prediction models for Sources A and B were completed, showing an acceptable error margin of ± 0.5 °C between actual and predicted values. Next, the blend winter specification of LPG was calculated using flow rates and actual spec values from Sources A, B, and C. Source C’s operational parameters were not considered due to its negligible flow rate, but its light-end composition significantly impacted vapor pressure (Figure 8).

While the predicted blend values aligned with actual values overall, discrepancies arose with predicted values generally being higher. This was likely due to underestimating the effect of Source C’s high concentration of light ends, which affected winter spec more than expected. Despite this, the model is reliable, as it errs on the safe side, ensuring the LPG product remains within spec expectations.

### 4.3 Prediction of LPG C_5_

**Figure 9:**
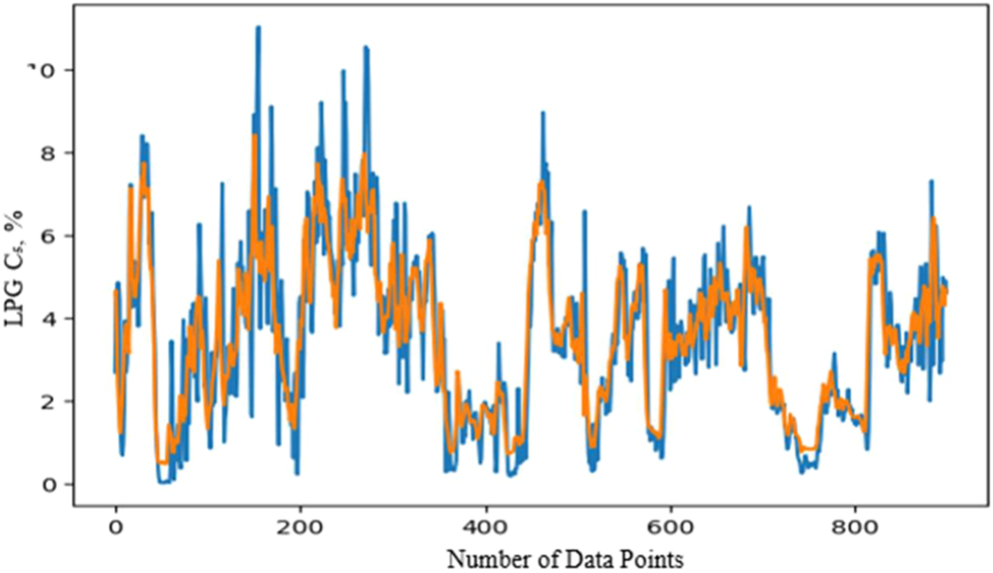
Blend LPG C_5_ specification: actual and predicted values.

Operational data for LPG from Source A, including six parameters and actual C_5_ values, was uploaded for analysis. The data set covered one year with an 8 h time lag between each entry. The data was then split into test, train, and validation sets to compare actual and predicted values over both short and long terms. Using TensorFlow’s Keras framework, relevant libraries were added, along with an optimizer and model checkpoints like MSE. A sequential prediction model was designed with 64 LSTM neurons and Dense layers. Next, prediction of C_5_ values from source B is carried out. First, data set is uploaded where, five parameters and actual values of C_5_ included. Data set is separated into train, test and validation data. For C_5_ model of LPG from source B is not perfect in compere to function A. this is the fact that actual values of C_5_ were also mainly between 0 and 1. Hence, due to quite a thick gap, the prediction was not too exact. However, the values of prediction also vary between these small gaps, hence the effect is small in this case. Blend LPG C_5_ specification is calculated with the train actual data from source A and B according to formula provided. Here also LPG from source C and its actual values of C_5_ also considered. Then, from predicted train values of C_5_ source A and B, blend prediction C_5_ is computed. The result of blend C_5_ values is given in Figure 9, where blue trend indicates actual, while orange trend indicates prediction model results.

### 4.4 LPG Propane Prediction

In the context of predicting propane content in LPG from Source A, it is logical to use the same set of operational parameters as those used for winter specification prediction, since propane plays a critical role in determining the LPG’s vapor pressure characteristics. Propane, being a light hydrocarbon, significantly impacts the winter specification, which is defined as the minimum temperature at which LPG maintains a desired vapor pressure. As the propane content increases, the vapor pressure of LPG rises, allowing it to maintain vaporization at lower temperatures. This results in a lower winter specification temperature, which is favorable for ensuring that the LPG remains usable in cold climates. Therefore, increasing the concentration of light hydrocarbons like propane will lower the winter specification temperature, making the product more suitable for winter conditions.

**Figure 10:**
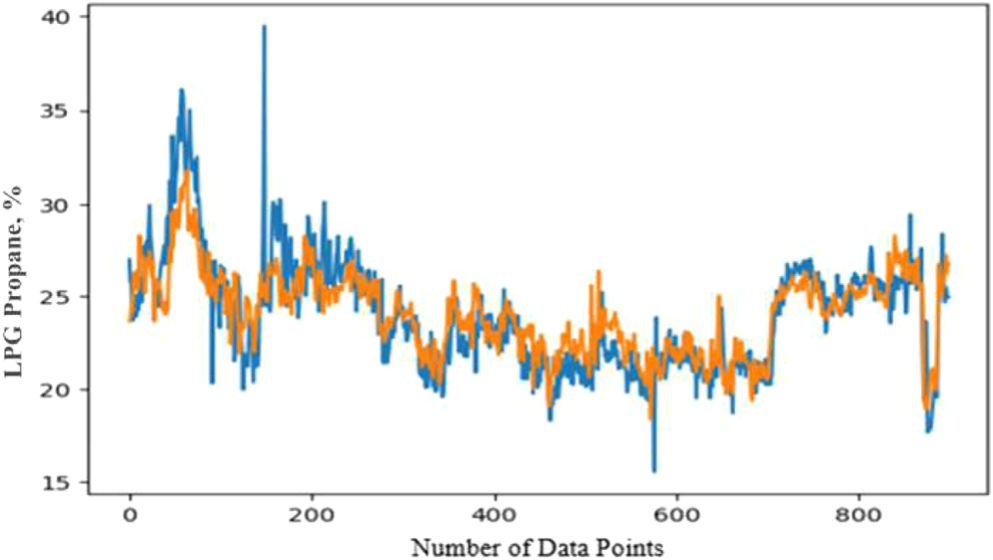
Blend LPG propane specification: actual and predicted values.

By leveraging the existing parameters that govern these behaviors, the model should be able to predict the propane content effectively while considering its influence on the winter specification. This approach helps ensure that LPG meets the desired vapor pressure criteria, preventing off-spec products and optimizing operational parameters. A data set with 15 parameters, including actual propane content values, was uploaded and split into test, train, and validation sets to evaluate both long- and short-term predictions. Using the TensorFlow Keras framework, a sequential LSTM model with 64 neurons and dense layers (8 and 1 neuron) was designed, with exponential activation functions. The propane prediction model for Source B was designed using a data set with nine parameters and actual propane content values over a one-year period with 8-h intervals. Source B LPG is rich in lighter hydrocarbons, resulting in higher propane content than Source A. However, the thermic process variations affecting propane content were not included in the data set. Different activation functions were tested to find the best solution by comparing MSE values. The exponential activation function yielded the lowest MSE (6.01), although the predicted and actual values did not perfectly align. Other activation functions performed poorly, with MSE values exceeding 300. Finally, the blend propane prediction model was created using the predicted values from Source A and B, while actual values were used for Source C. Despite the partial success of the Source B prediction, the blend prediction followed the actual values closely, as shown in Figure 10, with only a small gap between actual and predicted values at the beginning of the data set.

### 4.5 LPG Sulfur Prediction

**Figure 11:**
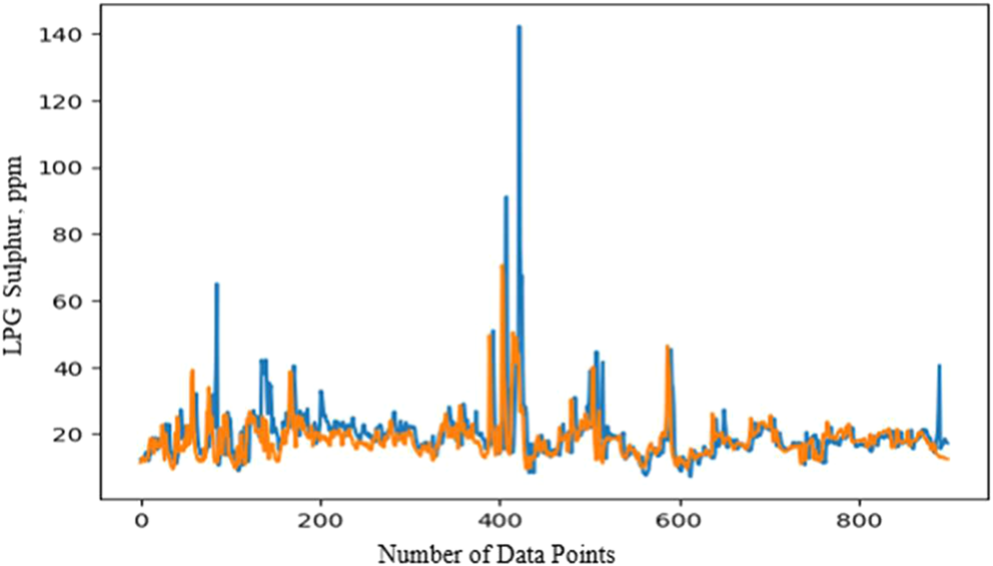
Blend LPG sulfur specification: actual and predicted values.

The LPG Sulfur prediction model starts by selecting operational parameters for production from Sources A, B, and C. A data set containing 27 parameters, including operational parameters and downstream data related to source A. Sulfur content is influenced not only by operational factors but also by crude composition. The data was split into test, train, and validation sets to predict both short and long-term trends. For prediction, the TensorFlow library from Keras was used with a sequential model. After testing different activation functions, the dense model with an exponential activation function was found to be the most effective for predicting LPG Sulfur from Source A. The long-term prediction closely followed actual values, and the short-term test prediction also matched well, except for one instance where Sulfur exceeded specifications. For source B, a similar approach was used, with 30 operational parameters over a one-year data set. The model was designed the same way, as Sulfur control is relatively consistent across sources. Predictions for source B also aligned closely with actual values for both the train and test data sets. Finally, the blend Sulfur specification was calculated by combining predictions from sources A and B. The results for the blend prediction, based on both actual and predicted values, are shown in Figure 11.

### 4.6 Optimizing Operational Parameters

In previous sections, each specification was optimized independently; however, some operational parameters serve multiple specifications, complicating their optimization. To address this, a single Particle Swarm Optimization (PSO) function was developed to optimize all LPG specifications collectively. This function combines the fitness functions for all specifications, optimizing 24 parameters simultaneously. To organize the optimization process, each specification and the related operational parameters are investigated.

Each LPG specification under consideration was individually evaluated to determine the operational parameter selections. Subsequently, these specifications, along with others subjected to a similar process, were synchronized and entered the optimization phase collectively. Each specification can also be optimized individually, making it possible to observe the isolated effects of changes in operational parameters. However, the presence of operational parameters that exert varying magnitudes and directions of influence on different specifications necessitates a holistic optimization model. In this context, the proposed approach ensures comprehensive integration and alignment of these parameters for optimal outcomes. The integration process involves incorporating all operational parameters into a single fitness function, as outlined in eq 5. This fitness function is then iteratively minimized to achieve optimal results.

To illustrate this process on a sample specification, sulfur has been selected as an example with 14 parameters, which were used both in the prediction process and could be manipulated. These parameters include the caustic flow rate, caustic properties, column operating conditions, and LPG flow rates from each source. Prediction models for LPG sulfur from Sources A, B, and the blend were then developed and uploaded. Once the parameters and models for sulfur optimization were uploaded, that part of the fitness function was defined. The European standard specifies a maximum LPG sulfur content of 30 ppm. Therefore, the fitness function can be designed to ensure the maximum sulfur value does not exceed 29 ppm during optimization. To calculate the fitness, a scale was assigned to each parameter. Scale values were defined based on their importance and range of variation, with all parameters normalized to a range of 0 to 1. Next, bounds were defined for the selected parameters. In PSO, defining bounds is critical to ensure that the search process remains within a feasible and valid region of the solution space. Bounds should be broad enough to allow sufficient exploration but narrow enough to maintain feasibility and avoid inefficient searches in irrelevant areas. For instance, one parameter used in the optimization of LPG sulfur is the excess oxygen content. Given that the maximum oxygen content in air is 21%, the upper bound for this parameter was set at 21%. However, minimizing oxygen content reduces caustic regeneration, so the lower bound for this parameter was set at 12%. This demonstrates the necessity of applying theoretical knowledge when defining bounds, as the feasibility and success of optimization depend heavily on these constraints.

When the fitness function is calculated, it integrates operational parameters that are both used in the prediction process and manipulable, contributing to the overall fitness score. The fitness function’s weights or scaling factors determine the relative importance of each parameter’s contribution to the score. It is specifically designed to minimize the error or discrepancy between the prediction model’s output and the reference value of 29 ppm. Each term in the fitness function computes the absolute difference between a parameter and its corresponding reference value. Minimizing the fitness function reduces the overall error and aligns the model’s output closer to the desired reference values. By incorporating parameters into the fitness function, the optimization algorithm explores various parameter combinations to identify the optimal set that minimizes the error. Therefore, this comprehensive search optimizes both the prediction model and the parameter values simultaneously. Including parameters in the fitness function provides flexibility and control over the optimization process, enabling fine-tuning of the model and its associated parameters to achieve the best possible alignment with the reference values or desired outcomes.

The same process is followed for other specifications, with the operational parameters and, consequently, the components of the fitness function being integrated into the holistic model. Once all components are finalized, predictions are generated based on the inputs. Following this phase, the optimization process determines the input values required to minimize the average error of the predictions. Additionally, the operational parameters that remain within the specified ranges are calculated to ensure compliance and feasibility. Finally, by processing all selected specifications and their input parameters within the same integrated model, the values presented in Table 1 were obtained. The usability of these values was also validated by process supervisors.

**Table 1:** LPG Specifications: Overall Variable Optimization

variables	UOM	scale	RUN1	RUN2	RUN3	RUN4	RUN5
stabilizer top T	°C	0.8	44.90	42.40	47.70	41.70	44.10
stabilizer top P	bar	0.8	5.00	5.10	5.30	5.10	5.10
debut top P	bar	0.6	11.40	10.30	11.70	10.40	10.60
debut top T	°C	0.6	53.90	51.70	51.00	51.60	54.50
caustic circulation rate A	Sm^3^/h	0.5	1.10	1.80	2.00	2.10	1.20
excess O_2_ A	%	0.5	14.80	10.40	10.60	19.90	13.20
caustic mercaptide A	ppm	0.5	96.40	53.00	63.80	97.00	21.90
caustic spent A	%	0.5	19.60	7.10	11.40	17.60	9.60
source A- LPG flow	ton/h	0.6	19.00	19.50	19.40	19.10	19.60
reflux	Sm^3^/h	0.5	99.50	21.30	37.00	89.40	71.60
caustic rate B	Sm^3^/h	0.5	2.00	1.20	1.60	0.80	1.10
caustic mercaptide B	ppm	0.5	65.00	147.00	102.00	69.10	114.70
caustic spent B	%	0.5	17.20	15.40	6.70	7.90	8.70
source A- LPG flow	ton/h	0.3	9.60	9.40	9.80	9.90	9.90
reflux flow	Sm^3^/h	0.5	94.30	78.96	82.90	91.60	63.20
absorber temperature	°C	0.5	48.30	52.30	58.00	53.90	55.40
column overhead Op	%	0.5	16.00	28.20	12.00	13.80	17.10
lean oil	Sm^3^/h	0.5	53.00	22.40	44.7	52.60	32.10
exchanger outlet temperature	°C	0.6	59.20	40.70	43.60	49.30	44.40
naphtha recycle	Sm^3^/h	0.5	80.90	103.40	97.60	108.70	96.80
stripper temperature	°C	0.5	158.9	158.80	159.00	153.00	151.70
sponge oil	Sm^3^/h	0.5	60.60	41.90	66.00	51.40	69.70
OUT of F-001 Pass-1	°C	0.7	503.90	495.00	502.10	505.00	501.70
OUT of F-002 Pass-2	°C	0.7	500.16	505.00	502.00	497.00	503.80
**sulfur prediction**	**ppm**	**1.0**	**22.60**	**26.80**	**13.10**	**17.80**	**12.10**
**C**_**5**_**prediction**	**%**	**0.2**	**5.10**	**0.60**	**6.10**	**3.60**	**3.40**
**winter spec prediction**	**°C**	**0.8**	**−7.80**	**−7.20**	**−9.30**	**−6.80**	**−6.20**
**propane prediction**	**%**	**0.5**	**20.30**	**23.80**	**22.20**	**23.10**	**21.20**
**blend LPG 1.3 butadiene**	**%**	**0.5**	**0.991**	**0.991**	**0.994**	**0.101**	**0.991**

Table 1 presents the results of this overall variable optimization, highlighting multiple viable solutions within predefined limits. The selection of the ″best″ solution is contingent upon specific constraints. For instance, if the operating pressure in in the Stabilizer must remain fixed at 5.3 kg/cm^2^g due to equipment or safety restrictions, the optimization must identify effective solutions within this fixed pressure. Particle Lean Oil shows that as the number of this variable increases, winter spec is minimized drastically. On the other hand, absorber temperature negatively increases effects to winter spec of LPG. Similarly, if the outlet temperature of a heat exchanger cannot drop below 50 °C due to operational challenges, the optimization process must ensure all solutions comply with this constraint.

The heater temperature is maximized to maintain the LPG 1.3-butadiene specification at its optimal upper limit of 0.1, even though the heater temperature range, defined between 450 and 510 °C, is relatively broad. This outcome arises from the fact that, based on the original actual data, the heater temperature predominantly remains below 504 °C. Consequently, the model does not extrapolate beyond this temperature threshold.

As the optimization model is refined and more runs are performed, the solutions converge toward a narrower range, suggesting that the optimization process effectively approaches the true optimal range given the constraints. Table 1 includes only those results that exhibit meaningful differences, representing feasible solutions under varying conditions. In conclusion, despite the limitations on manipulating certain operating parameters, the overall optimization indicates that the system can still operate reliably within desired specifications, demonstrating effective guidance toward optimal performance within defined limits.

## 5 Conclusions

This study has substantially contributed to by integrating advanced methodologies, specifically LSTM prediction and PSO optimization, to address the intricate challenges in predicting and optimizing LPG specifications. The fusion of LSTM’s temporal awareness with PSO’s fine-tuning capabilities has yielded a robust solution for the petrochemical industry. While there is no dedicated research on employing LSTM prediction and PSO optimization specifically for LPG product specifications, previous studies have demonstrated the integration of predictive modeling and optimization across different industries. These earlier investigations offer a foundation for exploring the potential benefits of LSTM and PSO in enhancing LPG production processes. Drawing insights from related research, this novel approach has the potential to improve the efficiency of LPG production and contribute to advancing our knowledge of optimizing processes within the refining industry. The utilization of PSO to optimize operational parameters following LSTM predictions has contributed to the precision of operational conditions. This has led to a notable advancement in consistently producing LPG products within specified parameters, minimizing variations, and ensuring adherence to quality standards.

The model is designed without incorporating calculations specific to the characteristics of the data set. The chosen deep learning approach is capable of effectively capturing the underlying dynamics of the data, provided it is sufficiently comprehensive. Additionally, the optimization technique is robust and unaffected by data-specific conditions. Consequently, the proposed framework is versatile and can be applied across various products and production processes. The one-year data set encompasses all known potential scenarios, and in the case of an unforeseen condition, the findings can be promptly updated by integrating the new data. This model has demonstrated suitability for deployment within the refinery, offering robust decision-support capabilities.

The practical implications of these findings are substantial. With the optioned results, it is estimated that off-spec production can be reduced from 10% to a minimum by applying deep learning techniques to predict product specifications and process optimization accordingly. This reduction has dual benefits as it lowers energy and chemical usage, contributes to cost savings, and significantly decreases the man-hours required for manual adjustments and quality control. Cost savings were estimated at around 800k USD annually as off-spec LPG is directed to the fuel gas system or reprocessed. The reduction in manual interventions streamlines operations, making them more efficient and less labor-intensive. By proactively addressing sources of off-specification, the optimization model helps to enhance overall operational efficiency and product quality. This innovative approach emphasized the critical role of digital tools in optimizing LPG production processes within the petrochemical industry, driving operational efficiency and cost-effectiveness.^49^ Beyond the technical implications, the managerial impact of implementing such a system is noteworthy. A decision support system was introduced incorporating scenario analyses to enable managers to make informed decisions, considering various possibilities and potential outcomes. This can result in more strategic and proactive decision-making, aligning production processes with organizational goals. A faster optimization method aligns with the need for efficiency in the dynamic field of chemical production. However, it is crucial to note that the adaptiveness of the system, particularly in the context of APC, might present challenges. Further exploration is required to optimize the system’s adaptability for real-time changes, ensuring its suitability for dynamic production environments. The strongest aspect of this study lies in its ability to go beyond merely predicting potential future issues based on commonly monitored data. While the prediction model provides foresight, process supervisors are then prompted to consider and plan preventive measures to avert such situations. Among these measures, adjusting operational parameters often becomes necessary. However, under the pressure of time constraints, identifying the optimal values for parameters that influence different specifications with varying magnitudes and directions can be a significant challenge. At this critical juncture, the proposed model’s optimization phase takes a step back from predictions and offers optimal operational parameters that avoid off-specification outcomes. This proactive capability effectively prevents undesirable scenarios stemming from parameter deviations, thereby enhancing the robustness of process management.

Looking forward to the future, research could address certain limitations in our study. We acknowledge that certain variables, such as the effects of crude oil feedstock, were not included. In particular, the challenges associated with crude feedstock could significantly impact the production rate of LPG, given that the amount of LPG generated varies with the type of crude oil. This variation is due to the lighter nature of LPG in comparison to other petroleum products. In this study, LPG flow rates are maintained stable for each source, but further development could explore the intricacies of crude oil feedstock challenges. Incorporating the dynamic effects of different crude oil types on LPG production rates could provide a more comprehensive understanding and improve the accuracy of predictive models. A crucial limitation to acknowledge is the exclusion of certain variables, especially the effects of crude oil feedstock, in this study. The simplification of assuming stable LPG rates for each source highlights the need for future research to delve deeper into the complexities of crude oil feedstock challenges and their impact on LPG production rates. This would contribute to a more nuanced and accurate representation of the real-world production environment. In conclusion, while the proposed methodology shows promise for LPG production optimization, there are opportunities for further research to enhance the model’s scope and accuracy, particularly in addressing the challenges associated with crude oil feedstock variations. Additionally, considering the limitations, this study serves as a stepping-stone for future investigations that could provide more comprehensive insights into the intricacies of LPG production. The model’s adaptability to real-time changes in operational parameters remains a challenge, particularly in dynamic production environments. Future research may address these limitations to improve adaptability and accuracy.
